# Automated microscopy and pH test for diagnosis of vaginitis – the end of empiricism?

**DOI:** 10.1038/s41746-023-00850-7

**Published:** 2023-09-06

**Authors:** J. D. Sobel

**Affiliations:** Wayne State University School of Medicine, Detroit, MI USA

**Keywords:** Physical examination, Reproductive signs and symptoms

## Abstract

At a time of diminishing clinical skills and diagnostic endeavours for clinicians worldwide, a method of rapid automated microscopy coupled with pH measurement is introduced with early clinical success results in women with common vulvovaginal symptoms facilitating rapid diagnosis and enhanced therapeutic measures.

There is a unanimous verdict regarding the universal phenomenon of decline in medical practitioners traditional clinical and hence diagnostic skills. To the rescue has arrived cutting edge molecular diagnostic techniques now widely available commercially, providing increased diagnostic sensitivity but not specificity with generated detailed information often exceeding the practitioner’s ability to assimilate the sophisticated molecular answers. This situation is identical to that seen decades ago when new cultures methods introduced annually increasingly were able to identify microorganisms previously “uncultivatable”, but never enhanced causality, i.e. establishing a causal role for the microorganism identified. Needless to say the frequency of women presenting with lower genital tract symptoms has only increased, at a time when clinical diagnostic skills, expressed in taking an adequate history and performing a competent physical examination, has dramatically declined. Practitioners increasingly descend into the world of empiricism or guessing and are thrust into obtaining readily available expensive PCR-based commercial tests that often do not facilitate diagnosis. Why not?—Lower genital tract symptoms in women are caused by a wide variety of causes, only a fraction of which are microbial or infectious in origin. Identifying these microorganisms by molecular methods clearly is now possible and superior to older culture techniques and use of traditional point of care rapid techniques including microscopy and pH measurement. Frequent causes of symptoms include hormonal factors, inflammation or relate to immune mechanisms as well as those causes that originate outside the vagina.

Modern molecular methods fail in the role of identifying or suggesting non-microbial pathogenesis, and even when organisms including pathogens are identified, they fail to establish causality. So how do we identify non-infectious causes and prove causation when organisms are now detected. Several steps are involved in identifying a diagnostic option. First a thorough personal history is followed by skilled genital physical examination and then the missing link viz vaginal pH measurement and skilled microscopy, (saline and potassium hydroxide). Unfortunately, pH measurement, which only takes seconds, is rarely done and microscopy is performed in less than a third of patients. Why not?—Too complex to answer in full, but practitioners routinely graduate from training programs without skills or discipline, and never schooled in appreciating the relevance of diagnosis. Once in practice, no time is allowed for microscopy or facilities are unavailable to implement this step^1^. Conclusion—delayed diagnosis and sending out molecular tests. After decades of desperate pleas by vaginitis specialists, this situation is irreversible and intractable. Preaching to practitioners to perform microscopy has not achieved one iota of success. Accordingly, molecular tests in spite of their minimal advantage are unfortunately still better than nothing^2^.

Now along comes a novel modern instrument performing automated microscopy and pH measurement but also integrated and part of a software logic facilitating the interpretation of the findings of this new instrument^3^. The arrival of this new approach is a solution to the missing components of the diagnostic process but in no way replaces the need for obtaining a history, performing a thorough physical examination and still occasionally performing focused additional nonpoint of care laboratory tests e.g. Candida species identification. The results presented in this early study reflect the experience of a single experienced clinician and success needs to be replicated in real life situations, including populations where all relevant cell pathogens including T.vaginalis are represented. Early results are impressive and offer an optimistic approach to its future use. This new approach reflects the importance of restoring microscopy in gaining insight into a complex vaginal microbiome at a time that clinicians no longer have the skills to benefit from using the traditional instrument. However, what is proposed this is more than mere microscopy. It is the accompanying implicit software logic that provides artificial intelligence in interpreting the findings revealed.

No doubt with wider use, diagnostic failures or dilemmas will emerge, however solutions and improvements will follow in turn. One also needs to recognise that symptomatic women present frequently with unusual causes of common lower genital tract symptoms often requiring frequent visits and evaluation. Yet others present with mixed infections and multiple simultaneous underlying causes, ensuring that diagnosis will always be a challenge. The proposed instrument offers a reasonable logical solution to replacing a vanishing skill without resorting to next generation sequencing and concepts beyond the understanding of clinicians.
